# Assessment of Nutritional Status of Infants Living in Arsenic-Contaminated Areas in Bangladesh and Its Association with Arsenic Exposure

**DOI:** 10.3390/ijerph15010057

**Published:** 2018-01-02

**Authors:** Abul Hasnat Milton, John Attia, Mohammad Alauddin, Mark McEvoy, Patrick McElduff, Sumaira Hussain, Ayesha Akhter, Shahnaz Akter, M. Munirul Islam, AM Shamsir Ahmed, Vasu Iyengar, Md Rafiqul Islam

**Affiliations:** 1 Centre for Clinical Epidemiology & Biostatistics (CCEB), School of Medicine and Public Health, Faculty of Health, The University of Newcastle, Kookaburra Close, New Lambton Heights, NSW 2305, Australia; john.attia@newcastle.edu.au (J.A.); mark.mcevoy@newcastle.edu.au (M.M.); patrick.mcelduff@newcastle.edu.au (P.M.); sumaira.87@gmail.com (S.H.); MdRafiqul.Islam@gvhealth.org.au (M.R.I.); 2 Department of Chemistry, Wagner College, 1 Campus Road, Staten Island, NY 10301, USA; malauddi@wagner.edu; 3 Goulburn Valley Health, Graham Street, Shepparton, VIC 3630, Australia; akhterayesha2001@gmail.com (A.A.); vasudha.iyengar@gvhealth.org.au (V.I.); 4 Department of Paediatrics, Institute of Child and Mother Health, Matuail, Dhaka 1212, Bangladesh; shahnaz.akter@uon.edu.au; 5 International Centre for Diarrhoeal Diseases Research, Mohakhali, Dhaka 1212, Bangladesh; mislam@icddrb.org (M.M.I.); shamsir.ahmed@menzies.edu.au (A.S.A.); 6 Department of Rural Health, University of Melbourne, Graham Street, Shepparton, VIC 3630, Australia; 7 School of Health and Social Development, Deakin University, VIC 3125, Australia

**Keywords:** arsenic, Bangladesh, drinking water, infants, children, malnutrition, underweight, stunting, wasting

## Abstract

Data is scarce on early life exposure to arsenic and its association with malnutrition during infancy. This study followed the nutritional status of a cohort of 120 infants from birth to 9 months of age in an arsenic contaminated area in Bangladesh. Anthropometric data was collected at 3, 6 and 9 months of the infant’s age for nutritional assessment whereas arsenic exposure level was assessed via tube well drinking water arsenic concentration at the initiation of the study. Weight and height measurements were converted to *Z*-scores of weight for age (WAZ-underweight), height for age (HAZ-stunting), weight for height (WHZ-wasting) for children by comparing with WHO growth standard. Arsenic exposure levels were categorized as <50 μg/L and ≥50 μg/L. Stunting rates (<−2 SD) were 10% at 3 months and 44% at both 6 and 9 months. Wasting rates (<−2 SD) were 23.3% at 3 months and underweight rates (<−2 SD) were 25% and 10% at 3 and 6 months of age, respectively. There was a significant association of stunting with household drinking water arsenic exposure ≥50 μg/L at age of 9 months (*p* = 0.009). Except for stunting at 9 months of age, we did not find any significant changes in other nutritional indices over time or with levels of household arsenic exposure in this study. Our study suggests no association between household arsenic exposure and under-nutrition during infancy; with limiting factors being small sample size and short follow-up. Difference in stunting at 9 months by arsenic exposure at ≥50 μg/L might be a statistical incongruity. Further longitudinal studies are warranted to establish any association.

## 1. Introduction

In Bangladesh, the prevalence of any form of under-nutrition among under-5 children is one of the highest in the world, with 43% stunted, 41% underweight and 17% moderate to severely wasted [1]. Among young children in Bangladesh, high malnutrition can be as a consequence of intrauterine growth retardation and postnatal growth faltering [2,3]. Childhood under-nutrition could be even higher in the areas where drinking water is contaminated with arsenic compared to other areas and warrants an evaluation.

Intrauterine growth retardation is related to many known and unknown maternal and environmental causes including maternal exposure to arsenic. Higher concentration of cadmium, lead and arsenic in placenta are associated with low birth weight [4] while maternal exposure to arsenic alone is related with reduced birth weight [5,6]. Also, suppression of Insulin-Like Growth Factor 1 (IGF-1) due to prenatal exposure to arsenic contaminated water partly causes growth impairment in children [7]. Poor postnatal growth is likely to be due to many factors such as, lack of exclusive breastfeeding, inappropriate complementary feeding and micronutrient intake, and a high prevalence of enteric infections along with poor hygiene practices [8].

Despite high prevalence of breastfeeding in Bangladesh, exclusive breastfeeding (EBF) in the first six months of life is not common [9,10] and the rate of EBF has in fact, declined over time [9,10]. In some families, it is quite common to discard colostrums and giving prelacteal foods to newborns [11]; furthermore, onset of breastfeeding is delayed by more than 24 h after birth [12]. The results of one study reveal that although 100% of mothers breastfed their infants from birth to one years of age, they added inappropriately prepared breast milk substitutes and starchy foods to the diets of 60% infants by three months and to 80% by five months of age [13], which may have led to higher rates of undernutrition during infancy.

EBF is important in the context of arsenic in the terms that even in women exposed to high levels of arsenic in drinking water, the breast acts as a filter and only a small amount of arsenic is secreted in breast milk [14]. However, long term effects may still occur in infants with low dose exposure of arsenic [15]. When breastfeeding is exclusive, infants are unlikely to be exposed to arsenic by other routes of exposure such as external foods, drinking water and foods made at home using arsenic contaminated water.

In known arsenic contaminated areas, infant exposure to inorganic arsenic might be higher but its impact on nutritional status has seldom been investigated globally. A study in Bangladesh is among the few that have assessed the impact of arsenic exposure on infant anthropometrics, finding that arsenic exposure was associated with lower body length and weight [16]. A study in Bangladesh evaluated malnutrition in older children by body mass index (BMI) and its association with arsenic, [17] while another study observed a negative effect of arsenic contamination on the acute nutritional status of children aged 7–14 years [18]. More recent studies also reported lower nutritional status among children in Bangladesh who were exposed to arsenic contaminated water [19,20]. Considering the paucity of arsenic related infantile malnutrition data and distinct ways of arsenic exposure in infants, this study evaluates the nutritional status of a cohort of infants living in arsenic contaminated areas in Bangladesh.

## 2. Materials and Methods

This longitudinal study was conducted during the period of March 2011 and March 2012 in an arsenic contaminated upazilla (sub-district) of Bangladesh. A total of 120 mother-infant pairs were recruited prospectively. Apparently healthy newborn-mother pairs, who planned to live in the study area for a period of at least 12 months and willing to provide required information and body measurements were considered eligible and invited to participate in the study with written informed consent. This study collected anthropometric data at 3, 6 and 9 months of infants’ age along with other necessary information such as socio-economic and dietary information.

### 2.1. Data Collection Procedure

A list of potential pregnant mothers and mother-newborn pairs in the selected unions (second smallest geographical administrative unit) was prepared and supplied by the upazilla family planning staffs under the Ministry of Health and Family Welfare, Government of Bangladesh. Households from the list in the selected unions were screened by three trained Field Research Assistants to ascertain who gave birth in the previous one month and who would give birth in the following three months from the day of household visit by the Field Research Assistants. Objectives and expectations of the study were thoroughly explained to the mothers of a newborn and the information sheet was read out to them during the household visits. At the time of initial contact with the participants and during subsequent household visits, the Field Research Assistants advised the household members not to drink or use for cooking water retrieved from tube wells that were marked with red color. The red markings signified tube wells contaminated with high concentration of arsenic (≥50 μg/L) which is beyond the WHO (World Health Organization) safety limit for drinking water in Bangladesh. The study information sheet was then left with the eligible participants for 48 h after which they were contacted either via mobile phone or by household visit for obtaining written informed consent from the infant’s guardian. Subsequently, Field Research Assistants collected baseline and follow-up information as well as anthropometric measurement data of infants at 3, 6 and 9 months of age.

### 2.2. Exposure Measurement

For each participating mother-baby pair’s household, a single tube well water measurement was used to characterize current arsenic exposure at the time of baseline data collection. Drinking water samples from the selected tube wells were transferred and analyzed following standard procedure [20,21]. Water arsenic analyses were performed at a Water Quality Testing Laboratory in the United States of America (USA) using flow injection-hydride generation atomic absorption spectrometry (FIHG-AAS) [21]. Minimum detection level of arsenic by this method is 3 μg/L.

### 2.3. Other Information

Interviewers collected information by face to face interview using a structured questionnaire on socio-demographics, drinking, bathing, washing, use of cooking water and history of water use. Information on postnatal food and drink intake was also collected.

### 2.4. Outcome Measurement

Trained field workers took anthropometric measurements such as weight and length of all participating infants at 3, 6 and 9 months of age. Nutritional measurements were carried out following standard procedure [22]. Infant’s weight was the deductive outcome of combined mother-baby weight and the mother’s weight only. Weight was measured using a digital bathroom scale with a precision of 100 gm (TANITA Corporation, Tokyo, Japan). The weighing scales were standardized/calibrated with a one kg standard weight prior to each working day.

Child’s length was measured using a locally made length-board in which a metal tape is extended between a footplate and head bar. The mean of three consecutive measurements to the nearest 0.5 cm was recorded as the observed value.

Weight and height measurements were converted to *Z*-scores of weight for age (WAZ-underweight), height for age (HAZ-stunting), weight for height (WHZ-wasting) for children by comparing with WHO growth standard using WHO-Anthro 2006 software (version 3.2.2, WHO, Geneva, Switzerland).

### 2.5. Statistical Analysis

Data were analyzed using STATA version 11 (STATA Corporation, Texas, TX, USA). Frequency tables were prepared to assess distributions of continuous variables, check for missing data and out of range values. Water arsenic level was used to characterize arsenic exposure. Arsenic concentration was categorized as <50 μg/L and ≥50 μg/L and also as quartiles. We also calculated the household’s asset score using the principal component analysis.

*Z* scores of malnutrition were categorized as having underweight (weight-for-age *Z* score, WAZ <−2.00 SD); wasting (weight-for-height *Z* score, WHZ <−2.00 SD), stunting (height-for-age *Z* score, HAZ <−2.00 SD) or not having malnutrition (if WAZ, WHZ and HAZ ≥−2.00 SD). Stunting, wasting and under-nutrition status were then compared with the Bangladesh Demographic and Health Survey 2011 (BDHS 2011) childhood malnutrition data.

## 3. Results

We recruited a total of 120 mother-newborn pairs. Half of the participating infants were female. Most of the mothers (94%) were relatively young (<30 years) and none of them were illiterate, while 30% completed their secondary level of study. Nearly all (97%) of the mothers were housewives; 84% of mothers did not know about the birth weight of their newborn and 18% were unaware of the duration of their pregnancy. On the other hand, 34% of the fathers completed secondary education and all of them were employed. Average monthly household income was 81 US$ (±81, 1 US$ = 80 Taka of Bangladesh currency) and more than one third of the households earned <40 US$ per month (median monthly income US$53, IQR: US$37 to 87) (Table 1).

All of the households were using tube well water for drinking and about 12% of the tube wells were marked with red color and another 84% had no color mark. Very few of the households were using different means to purify their drinking water such as boiling (0.8%), use of disinfectant (3.3%), filtration in jar (1.7%) and simple filter (0.8%).

To understand the possible routes of arsenic exposure for infants, we collected breastfeeding and other liquid feeding information. Three fourth of the infants were introduced with breastfeeding immediately after or in the 1st hour of birth and 94% were fed colostrum. More than 82% of the infants were given different kinds of drinks/saps or formula milk and only 15% were not given anything other than breast milk within three days of birth while, 55% of the infants were given plain water within 24 h of birth. About 97% of the infants at 3 months, 93% at 6 months and 66% at 9 months of age were breastfed. Simultaneously, 37.5% of the infants at 3 months, 38.7% at 6 months and 16% at 9 months of age were offered pumped out breast milk in cups or spoons.

Among the study participants, overall stunting (<−2 SD) was 10% at 3 months and then 44% at 6 and 9 months of age. Severe wasting and underweight decreased as the infant grew older. Prevalence of underweight and wasting were the highest at 3 months of age. Overall wasting (<−2 SD) was 23.3% at 3 months and no infants were wasted at 6 and 9 months, while overall underweight infants (<−2 SD) were 25% and 10% at 3 and 6 months of age, respectively. None of the infants at 9 months of age were underweight. On the other hand, at the age of 6 and 9 months, no infant was suffering from any form of wasting.

The degree of malnutrition was categorized as having malnutrition (<−2 SD) or not having malnutrition (>−2 SD) every time anthropometric measurements were taken (Table 2) and compared with the relevant similar anthropometric data in the Bangladesh Demographic and Health Survey 2011 (BDHS 2011) [23]. We collected data at 3 intervals of the infants’ age (3, 6 and 9 months) while BDHS 2011 anthropometric data contains nutritional measurements at <6 months and 6–8 months of infants’ age. Therefore, a relevant statistical test to demonstrate differences in proportion was not performed; instead the prevalence data of our and BDHS 2011 studies were presented side by side in Table 3.

## 4. Discussion

Except for stunting at 9 months of age, we did not find any significant changes in other nutritional indices over time or with levels of household arsenic exposure in this study. A reasonable proportion of infants were underweight and wasted at 3 months of their age. Infants recovered from underweight and wasting at 6 and 9 months of age while the proportion of stunting (height for age *Z* score-HAZ) increased significantly at 6 months and remained static at 9 months of age. Interestingly, we observed a low proportion of malnourished infants in the households whose tube well water contained ≥50 μg/L of arsenic compared to the infants of the households whose tube-well water contained <50 μg/L of arsenic and the differences were mostly statistically insignificant except stunting at 9 months of age (Table 2). This might be due to the change of household’s drinking water sources following our advice of not drinking arsenic contaminated water at the initial stage of the study when the participants were recruited and drinking water samples were collected. Therefore, there was a lag period of varying arsenic exposure between drinking water sample collection at baseline and change of household drinking water sources for the duration of anthropometric measurements at 3, 6 and 9 months of infant’s age. Thereby, the observance of low prevalence of childhood malnutrition in the households whose tube well water contained ≥50 μg/L of arsenic was likely due to the participants having changed their drinking water sources. Despite this, there may be some impact on chronic malnutrition as the drinking water source had only been changed for a few months. Further data was not available detailing how many households changed their source of drinking water during the study and whether the inhabitants of the households whose tube well contained ≥50 μg/L of arsenic were truly exposed to that amount of arsenic via drinking water; serving as a potential limitation of the study. Additionally, the small sample size in this study could be another potential reason for not demonstrating any significant difference in most infant nutritional indicators with varying exposure to arsenic.

In terms of acute malnutrition (wasting and underweight), most of the infants were found well-nourished when compared with the Bangladesh Demographic and Health Survey data 2011. However, exact age matched nutritional comparison was not possible because the time points of data collection and age of the infants were different between our study and BDHS 2011 study (Table 3). Wasting (weight for height *Z* score-WHZ) reflects the current nutritional status (acute malnutrition), while underweight (weight for age *Z* score-WAZ) is not the right indicator for understanding any form of malnutrition e.g., acute or chronic [24]. In our study, we observed no or low wasting and underweight infants, which reflects low acute malnutrition among infants in the study area. These findings can be correlated with high rates of breastfeeding which result in low exposure to arsenic, especially at 3 and 6 months of child’s age. This may be the reason for observance of low prevalence of acute malnutrition in infants in the study area. These findings are in line with other studies conducted previously which demonstrated the beneficial effects of breastfeeding with childhood malnutrition and breastfeeding helping to achieve optimal growth in children [25,26,27].

Stunting, on the other hand, is the cumulative effect of chronic malnutrition and was noted to be high in our study. Recent improvements in dietary intake do not improve stunting remarkably [23]. Our data indicates a higher proportion of stunting in infants irrespective of their household’s arsenic exposure levels and stunting rate was higher when compared with BDHS 2011 data (Table 3). The reason for high rates of stunting in these households are unknown, however, maternal nutritional status could be helpful in explaining this. Chronic energy deficiency (BMI < 18.5 kg/m^2^) in the form of short stature is very common among women in rural Bangladesh. About 38.8% of the women in the rural areas of Bangladesh are chronic energy deficient [28] while 34% of the women of reproductive age in rural Bangladesh are malnourished [29]. It is evident that mothers’ short stature (<145 cm) are doubling the odds of having stunted children [30] and maternal stature are inversely associated with infant and childhood malnutrition [31,32]. Consequently, the higher prevalence of stunting in infants might be better explained by the higher prevalence of short stature in mothers in our study area rather than observing an association between arsenic exposure and stunting in children. Unfortunately, this explanation cannot be supported by our study as we do not have the mothers’ anthropometric data.

Malnutrition rates of Bangladesh are among the highest in the world, with stunting observed in half of the children less than 5 years of age because of poor dietary diversity [33]. No study was found to have reported an association with maternal or infant arsenic exposure to that of stunting in infants. One Bangladeshi study reported a weak association between maternal arsenic exposure and birth defects, but not with stunting and underweight among children [34]. Whereas another study reported lower body weight and length in association with postnatal arsenic exposure among female children from birth to two years of age [16]. The difference in stunting in infants from households with arsenic exposure from tube well water of <50 μg/L and ≥50 μg/L observed in our study of infants at 9 months of age could be a statistical anomaly and the small sample size of this study could be a factor influencing the lack of differences observed among infants in other nutritional indicators (wasting, underweight). We missed the opportunity to capture data on maternal and infant nutritional intake as that was beyond the scope of the study, which might have given more data on malnutrition issues.

## 5. Conclusions

Post-natal exposure to low amounts of arsenic, predominantly via breast milk, may not have an influence on infantile malnutrition. However, further longitudinal studies in infants are warranted to determine any association. Methods of exploring a dose-metered response to arsenic exposure in terms of nutrition would be valuable in determining appropriate public health policy recommendations.

## Figures and Tables

**Table 1:** Characteristics of infants and their families by arsenic concentration in drinking water of the household (*n* = 120).

Characteristics	Arsenic Concentration in Drinking Water		*p*-Value
	<50 μg/L (*n* = 80)	≥50 μg/L (*n* = 40)	
Mothers of infants age in year (Mean, ±SD)	23.9 ± 4.9 (80)	24.6 ± 4.1 (40)	0.4
Months of pregnancy child born (Mean, ±SD)	9.3 ± 0.56 (62)	9.2 ± 0.55 (36)	0.6
Infants gender (%):			0.7
Male	51.2 (41)	47.5 (19)	
Female	48.8 (39)	52.5 (21)	
Religion of the family (%):			0.2
Muslim	72.5 (58)	62.5 (25)	
Hindu	27.5 (22)	37.5 (15)	
Mother’s education (%):			0.3
No education	0.0 (0)	0.0 (0)	
Primary	63.7 (51)	47.5 (19)	
Secondary	26.3 (21)	37.5 (15)	
Post-secondary	2.5 (2)	2.5 (1)	
Can only write name	6.3 (5)	12.5 (5)	
Only religious education	1.2 (1)	0.0 (0)	
* Father’s education (%):			0.6
No education	0.0 (0)	0.0 (0)	
Primary	47.5 (38)	46.1 (18)	
Secondary	32.5 (26)	38.5 (15)	
Post-secondary	6.3 (5)	5.1 (2)	
Can only write name	13.7 (11)	10.3 (4)	
Only religious education	0.0 (0)	0.0 (0)	
Mother’s occupation (%):			0.6
Housewife	96.3 (77)	97.5 (39)	
Day labor	2.5 (2)	0.0 (0)	
Other	1.2 (1)	2.5 (1)	
Father’s occupation (%):			0.4
Day labor	21.3 (17)	17.5 (7)	
Farming own land	21.2 (17)	27.5 (11)	
Petty business	23.7 (19)	27.5 (11)	
Large business	3.8 (3)	7.5 (3)	
Others	28.8 (23)	7.5 (3)	
Not applicable (Father died/divorced)	1.2 (1)	0.0 (0)	
Monthly household income in Taka ^§^ (%):			0.1
1000–5000	62.5 (50)	47.5 (19)	
6000–10,000	27.5 (22)	37.5 (15)	
>10,000	10.0 (8)	15.0 (6)	
^Ɨ^ Number of mother‘s pregnancy (%):			
1–2	66.2 (53)	65.0 (26)	0.9
>2–4	23.8 (19)	25.0 (10)	
>4	8.8 (7)	10.0 (4)	
Socio-economic status; asset score (%):			0.5
Quintile 1	18.7 (15)	15.0 (6)	
Quintile 2	20.0 (16)	12.5 (5)	
Quintile 3	16.3 (13)	17.5 (7)	
Quintile 4	15.0 (12)	22.5 (9)	
Quintile 5	17.5 (14)	15.0 (6)	
Missing	12.5 (10)	17.5 (7)	

* 1 Information missing in arsenic category ≥50, ^Ɨ^ 1 Information missing in arsenic category <50; ^§^ 1 US$ = 80 Taka; column %.

**Table 2:** Prevalence of malnutrition (≤−2 SD) in infants at 3, 6 and 9 months of age by arsenic exposure (*n* = 120).

Indicators of Malnutrition	Age of the Infant								
	3 Months, *n* (%)			6 Months, *n* (%)			9 Months, *n* (%)		
	Arsenic <50 μg/L	Arsenic ≥50 μg/L	*p*-Value	Arsenic <50 μg/L	Arsenic ≥50 μg/L	*p*-Value	Arsenic <50 μg/L	Arsenic ≥50 μg/L	*p*-Value
Underweight (WAZ):									
<−2 SD	23 (28.8)	7 (17.5)	0.18	10 (12.5)	2 (5.0)	0.19	-	-	-
>−2 SD	57 (71.2)	33 (82.5)		70 (87.5)	38 (95.0)		80 (66.7)	40 (33.3)	
Stunting (HAZ):									
<−2 SD	9 (11.2)	3 (7.5)	0.52	40 (50.0)	13 (32.5)	0.06	42 (52.5)	11 (27.5)	0.009
>−2 SD	71 (88.8)	37 (92.5)		40 (50.0)	27 (67.5)		38 (47.5)	29 (72.5)	
Wasting (WHZ):									
<−2 SD	20 (25)	8 (20.0)	0.54	-	-	-	-	-	-
>−2 SD	60 (75)	32 (80.0)		80 (66.7)	40 (33.3)		80 (66.7)	40 (33.3)	

* Showing column percentage, unless otherwise indicated.

**Table 3:** Comparison of malnutrition data of infants in the study area with Bangladesh Demographic and Health Survey 2011 (BDHS 2011) data.

Indicators of Malnutrition	Age of the Infants			
	6 Months (Study Data) or <6 Months (BDHS Data)		9 Months (Study Data) or 6–8 Months (BDHS Data)	
	Study Data (*n* = 120)	BDHS 2011 (*n* = 695)	Study Data (*n* = 120)	BDHS 2011 (*n* = 403)
Underweight (WAZ):				
<−3 SD, %	0.0	4.4	0.0	5.7
<−2 SD, %	10.0	16.5	0.0	23.2
Mean Z score	−0.34	−1.0	0.51	−1.2
Stunting (HAZ):				
<−3 SD, %	25.0	4.6	35.8	5.4
≤−2 SD, %	44.2	18.0	44.2	17.4
Mean Z score	−1.79	−0.7	−2.2	−0.9
Wasting (WHZ):				
<−3 SD, %	0.0	6.3	0.0	4.3
<−2 SD, %	0.0	16.0	0.0	14.8
Mean *Z* score	1.2	−0.6	2.48	−0.7
